# Development of intellectual and scientific abilities through game-programming in Minecraft

**DOI:** 10.1007/s10639-022-10894-z

**Published:** 2022-02-08

**Authors:** Alessandro Bile

**Affiliations:** Department of Fundamental and Applied Sciences for Engineering, Sapienza Università di Roma, via A. Scarpa 16, Roma, Italy

**Keywords:** Technological learning, Digital education, Game-base learning, Programming, Minecraft education edition, Microsoft MakeCode

## Abstract

In recent years, the technological revolution has changed the way we see reality and interact with it. Inevitably, education and didactic planning have also had to deal with new technologies. Indeed, the presence of digital tools has radically changed people’s lives since childhood. Many educational realities have exploited this digital transformation to speed up and specialize learning, customizing study plans and type of software according to age groups. The activity of the Digital Education Lab is part of this context. It is a digital education school which uses the game software *Minecraft Education Edition* to teach its students the fundamental principles of computer science, geometry and mathematics. This article discusses learning key science concepts through game learning. The analysis carried out allows to see that students are facilitated in learning complex scientific concepts when these are shown through the game and can therefore be “experienced”. The learning of 186 students aged between 8 and 10, who are generally at the first approaches to the digital world, has been evaluated. To evaluate the acquisition of knowledge through these innovative methods, at the end of the didactic course we have administered anonymous tests through the Google classroom platform. The results show that learning through a game-software facilitates the learning of basic scientific information as well as fostering the capacity for interconnection and transversality.

## Introduction

The reality we are experiencing is dominated by the digitization process. This word, which is often seen by the more traditionalist educators in a negative sense, indicates a path of conversion from the analog world, dominated by continuity, to the world of computers, characterized by discretization. This translation is manifested through sequences of very fast electrical implusions which are considered as harmful to the mental development (both from the point of view of synaptic connections and of consciousness’ formation and therefore of identity) of the child. For this reason there is a need to educate today’s young people in the use of technology before educating them through technology. The transition to the digital world is spreading faster and faster and educational models need to keep up and evolve with this trend. The use of technology, however, could allow for an earlier and faster learning. The possibility of using computer tools allows in fact to introduce complicated abstract concepts into a game. In this way, different stimuli, sometimes more effective , are provided to the learners. Digital education is usually realized in two modalities: by the presence of an educator and by a remote online learning. Both of these realities have shown strengths and weaknesses, which vary according to the target age of the group. Moreover, the pandemic caused by the SARS-COVID19 infection highlighted these aspects (Dong et al. 2020). The public within 10 years of age show a strong need for physical contact with the teacher. The absence of the physical aspect determines a drastic decrease in attention. After this critical age, sufficient abstraction skills have developed to be able to learn also by the dialogue with a “screen”. In both cases, however, digital teaching has proven to be able to transmit basic knowledge through alternative methods, often more appreciated by young audiences (Bar-El David & Ringland 2020; Brown et. al 2021).

The relationship between students’ way of processing information plays a fundamental role in students’ learning (Asikainen & Gijbels 2017). The research group of (León et al. 2016) recognized that learning is directly dependent on many factors. Among these factors, the most important are the playful aspect (Zosh et al. 2017), the social one (learning with friends) and the satisfaction of the goals achieved. Their combination guarantees a very high attention span that allows a faster and at the same time more solid learning. Moreover the game represents a fundamental phase for the growth and development of individuals. Furthermore, it should not be overlooked that it contains the social-communicative aspect. Therefore, learning through playing, in this case thanks to a game software, favors the learning of concepts, often very difficult to understand according to traditional educational methods (Marton & Säljö 1976). The game facilitate to make concrete abstract concepts that especially younger students find them hard to imagine. The Digital Education Lab, a school of technological education based in Rome (Italy), has developed a method of teaching the fundamental concepts of mathematics, geometry and computer science (programming and not only) through the use of the game software *Minecraft Education Edition*. In this work we briefly report the teaching method of the Digital Education Lab and we discuss this type of educational path, analyzing the results of the tests that were administered at the end of the course. The tests are anonymous and are used to evaluate the effectiveness of teaching with respect to the various educational objectives that the school proposes. The teaching method of the Digital Education Lab aims to develop, in its students, the desire to learn and discover new things. This is why it exploits the games and social aspects, supporting collaborative training in problem solving and, ultimately, friendship.

## Minecraft Education Edition: A game software for the development of mental faculties

The fundamental objective, with which *Minecraft Education* was created, is to allow total freedom of exploration and creation (Klimovà et al. 2021, Carbonell-Carrera et al. 2021). Unless they are selected in the main menu modes, the game is emptied of goals (Peters et al. 2021, Pietarinen et al. 2018). This is one of the reasons why when the first version was released, no guide was published (Sajben et al. 2020). The game software reproduces *many different landscapes* (realities) where it is possible to play in single or multiplayer mode (Vesin et al. 2021, Wouters et al. 2013). The realities of Minecraft are based on cubic logic, which means that the smallest element in Minecraft is made up of a cube (or block) of a different material. Complex structures can be made by delivering multiple blocks into space. Players are free to move, make buildings and objects, freely choosing the type of material and in some contexts using chemistry to make them. From this brief introduction it is clear how the player is faced with the resolution of mathematical and geometry problems in the choice of the shape of the construction. Minecraft offers the possibility of playing through keyboard and mouse, through programming (available languages are MakeCode, python and JavaScript) or through both. Within the Digital Education Lab the following levels of player are distinguished on the basis of the skills possessed:

- User-friendly mode programmers: the player can simply choose the “block” (fundamental cubic brick of any type of material) and place it using the mouse.
- Beginner Programmers: the player can choose the quantity of blocks, the type of block and the position through the visual programming of MakeCode. In this case, the player can view the construction steps through a robot, Agent, to which the commands are given and which carries them out progressively.
- Intermediate Programmers: the player is able to write commands through the MakeCode language but without using the Agent. This procedure is suitable for a more advanced player because it does not allow the visualization of the various steps.
- Advanced Programmers: the game also allows the use of more advanced programming languages (pyhton and Javascript), which allow a wider realization but which are more difficult.

In this paper we will focus on the analysis of the teaching methods and the results achieved for the basic course of the Digital Education Lab. The basic course generally includes students aged between 8 and 10 and belonging to the User-friendly mode programmers and Beginner Programmers.

## Objectives sought in digital education

**Fig. 1:**
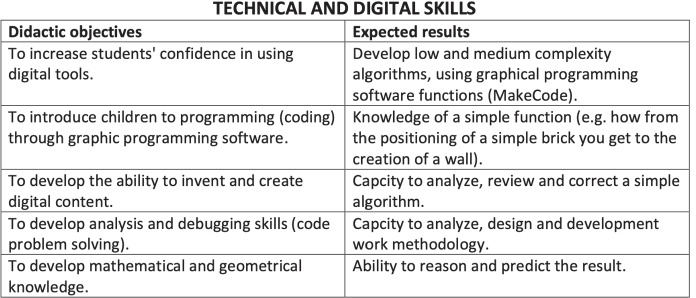
Summary of the technical and digital skills expected at the end of the course

Minecraft Education allows to shape an educational path designed to learn the basics of creative programming and become familiar with MakeCode, a graphical and block programming language specially developed by Microsoft. In the Digital Education Lab, the lessons are built in order to provide increasingly complex skills starting from simple concepts (Fig. 1).

**Fig. 2:**
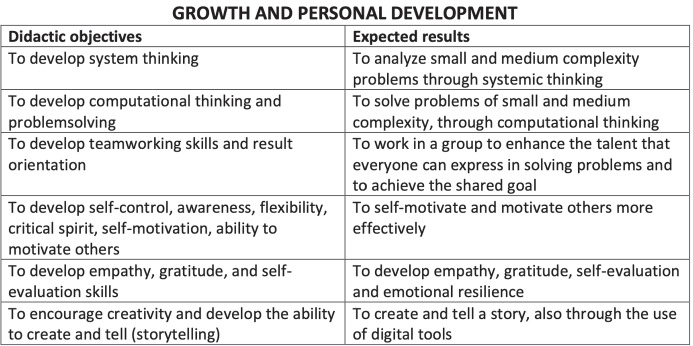
Summary of the growth and personal development expected at the end of the course

**Fig. 3:**
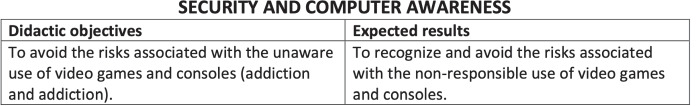
Summary of the security and computer awareness expected at the end of the course

The basic level gathers the user-friendly mode programmers and a first group of the beginner programmers. The educational path can be summarized in the following tables which aim to create and enhance qualities for intellectual development in children. The didactic objectives are organized in three preferential directions. First of all, teaching through a game software aims to develop knowledge and technical skills that can increase the student’s knowledge in a playful and more enjoyable way than traditional methods. The second fundamental aspect is the all-round growth of the person, Fig. 2: game-learning has shown great acquisition of awareness of children’s skills by helping to build the identity of the person. In addition, the multiplayer mode favors integration with others, favoring self-motivation and motivation of others, Fig. 2. Finally, the use of a software game for teaching favors the creation of opportunities for discussions on technological security, Fig. 3. Students who also follow this type of teaching seem to respond more consciously and autonomously to the risks associated with excessive use of electronic games.

## Didactic teaching method

The didactic approach used in the game-learning courses through Minecraft Education Edition achieves the didactic objectives introduced through the experientiality of the concepts before providing definitions or rules. Traditional teaching first provides assumptions and then, through them, proposes an investigation or an analysis. The teaching method of the Digital Education Lab is based on an inversion of this paradigm. From the experimental investigation we move on to the elaboration of categories and concepts. This favors a well-rounded understanding of the problem faced even when it comes to an abstract mathematical elaboration. Younger children in particular find it difficult to understand abstract definitions that are unable to fit into experience and reality. The opportunity to see the functioning first allows a concretization of the problem that is “experienced” in first person by the student.The great potential of this method is visible in the next section where the results of the teaching method will be investigated in all its nuances. Each course, which is divided into eight lessons of one and a half hours each, ends with a final test that is submitted to the students. In this test, whose structure is described in detail later, abstract definitions are also proposed that are never given during the lessons. Most students are able to recognize the accuracy of a definition or concept.

The educational method based on game-learning proposed by the Digital Education Lab is mainly divided into three fundamental bricks:

- complexity as a union of fundamental concepts;
- trial and error procedure;
- step visualization.

**Fig. 4:**
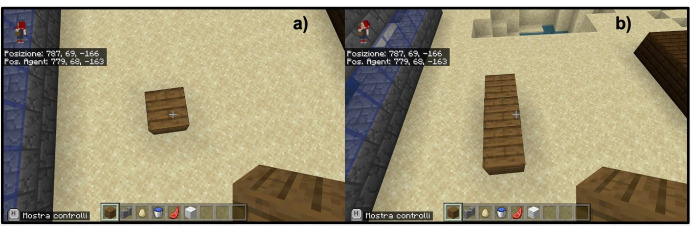
Example of didactic implementation of the item “Complexity as union of fundamental steps”, a) shows the single point (block), while b) shows the line as sequences of points (blocks)

**Fig. 5:**
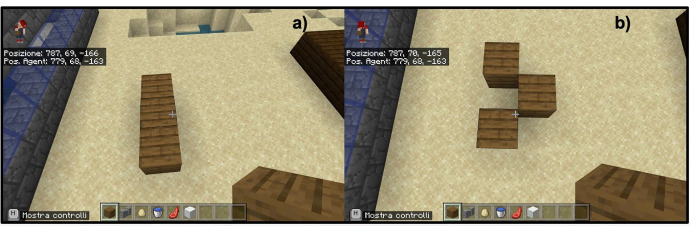
Example of didactic implementation of the item “Trial and Error” a) shows the right line, while b) shows the line shows the line with the center block in the wrong position

**Fig. 6:**
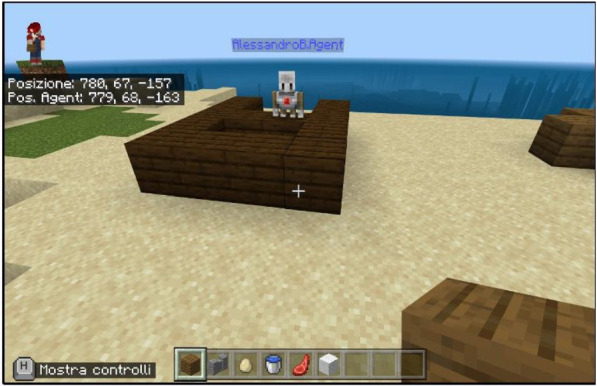
Example of didactic implementation of the item “Step Visualization”, Shows the Agent executing the given commands through the code

The first investigation strategy that is shown to students is to understand a complex problem by simplifying it into simpler components. Each structure that is proposed as food for thought can be seen as a function of the fundamental unit present in Minecraft or the single block (of any material). For example, students learn to see a line of blocks as a succession of points (blocks) arranged along a precise direction (Fig. 4). However, they are not told a priori what a straight line is. In this way they learn to see complex objects directly as interrelationships between simple parts. From the very first moment within the Minecraft Education Edition, students are taught that viewing each part of the project is essential to recognize any errors and act accordingly. Error does not become a negative quality but rather a necessary element for learning. Remaining in the basic example of the construction of a line of blocks, if the line is constructed crooked it is necessary to understand what happened and at what point in the reasoning something was wrong (Fig. 5). The last item at the base of the teaching method consists in visualizing each step of one’s code and therefore of one’s reasoning. This item is crucial for experimental learning especially for younger students. For them Minecraft Education Edition has thought of a robotic character (Agent) who carries out step by step each instruction given (Fig. 6).

## Research Methodology and analysis of tests

Digital education through game-learning overturns the educational paradigm of traditional teaching which first involves the transmission of theoretical knowledge and then its practical use. The Digital Education Lab thinks that, to facilitate the understanding of abstract concepts, it is important to concretize knowledge through practical experience. In this case it occurs through the playful component which, in addition to favoring a greater degree of attention, allows concentration to be kept high for a longer period of time (Zhang et al. 2021). Furthermore, students are led to autonomously elaborate an abstraction process that leads to the conceptual generalization of knowledge, as reported by the results of the tests that are administered at the end of the course. At the end of the educational path, students are given a test, built to evaluate the skills acquired. Therefore, the research on learning and the development of cognitive and scientific skills presented in this paper is based on two main questions:

- is it possible to transform mainly abstract computer, mathematical and geometric concepts into “livable” experiences?
- Are the students autonomously able to operate a procedure of generalization of experience to produce a scientific rule?

The tool used to analyze these aspects is a post-course test, built with a targeted articulation and consult the research topics introduced. At the beginning of the courses, no pre-course tests were administered as the students of the age group considered (8-10 years) have limited computer experience that does not require the use of programming. The starting level appears almost homogeneous. However, it is not excluded that, in future elaborations of this study, they will be able to benefit from pre-course tests appropriately constructed in accordance with the final test.

The pre-course test is developed to analyze the specific and transversal skills acquired through game-learning. For this reason it consists of theoretical and reasoning questions. The articulation of the test was designed to observe the students’ ability to solve practical problems, both simple and complex, and the ability to extract definitions and generalizations autonomously and independently. As already pointed out, during the courses the teachers try never to provide formal assertions but encourage curiosity in order to make the students able to elaborate their own conceptualization on the basis of practical experience. The test structure therefore also analyzes this aspect. The results reported in this work concern 186 students of the basic course who participated in the Coding course through Minecraft Education Edition in the year 2020-2021. Students are grouped into classes of four components. We have found that this number fosters a closer relationship with the teacher which results in more solid learning. Furthermore, the social dimension is also facilitated. The test is divided into nine questions, each of which is assigned a score of 1. Wrong answers are given a null score. This encourages students to think and not stop at the first difficulty.

Evaluation objectives aim to include four different learning areas, each of which is given a topic identification code. The initial acronym identifies the area of expertise being analyzed (INF, MAT, GEO) while the following number indicates specific aspects and increasing difficulties within the same area of expertise. The list below analyzes in detail the skills needed to be able to answer the questions correctly, based on the area of expertise and the level of difficulty.

- INF/0: Knowledge of basic technical informatic terminology.
- INF/1: Ability to translate the analyzed simple problem into computer language (Make Code).
- INF/2: Ability to translate the analyzed complex problem into computer language (Make Code).
- GEO/0: Knowledge of basic technical geometric terminology.
- GEO/1: Ability to analyze simple geometric shapes and to break them down into fundamental parts.
- GEO/2: Ability to analyze complex geometric shapes and conceive them as a union of simpler geometric shapes.
- MAT/0: Knowledge of basic technical mathematical terminology.
- MAT/1: Ability to schematize simple problems in mathematical form and to solve numerical calculations.

**Fig. 7:**
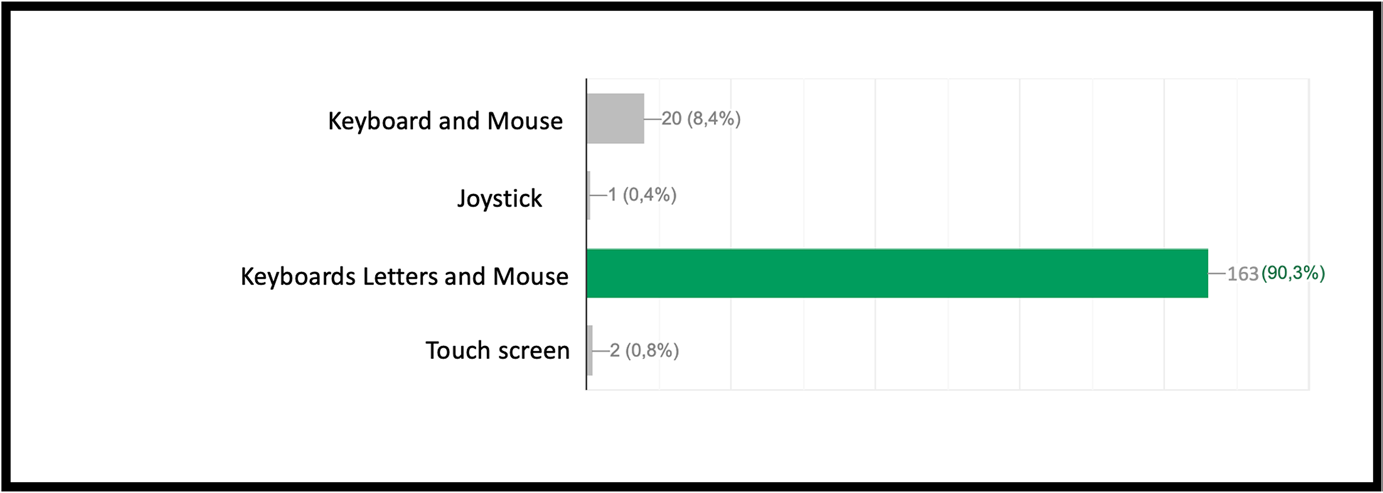
Score as a percentage of possible answers to the first question of the sector INF / 0 *“What are the commands to be able to play Minecraft on the PC”*

**Fig. 8:**
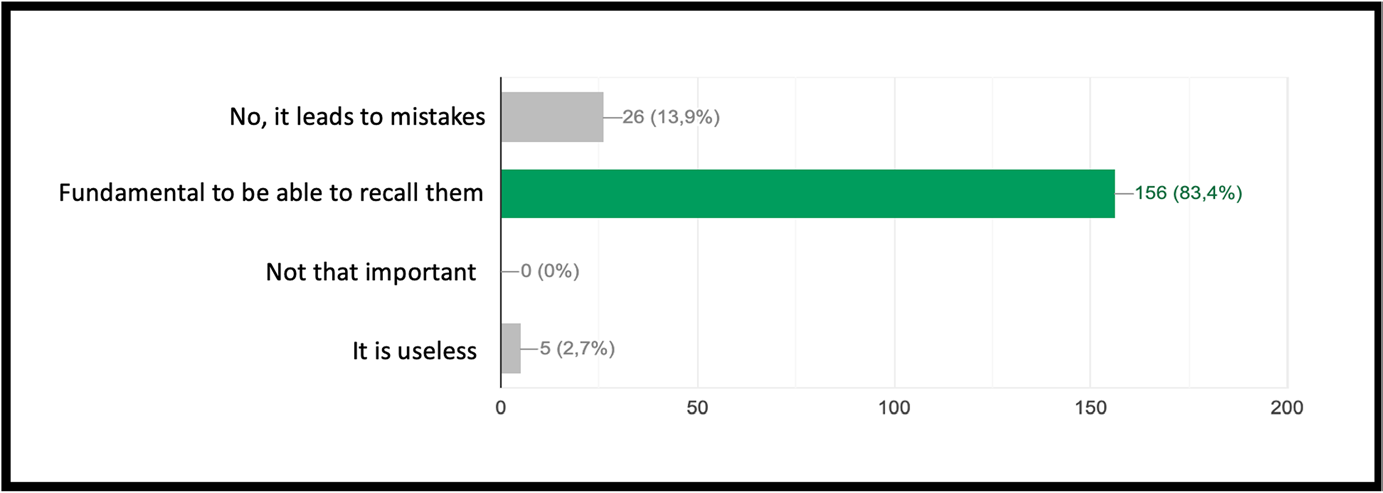
Score as a percentage of possible answers to the second question of the sector INF / 0 *“do you think it is useful to give names to the commands so that they are easier to understand?”*

**Fig. 9:**
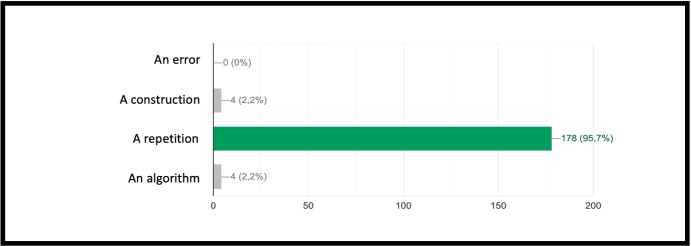
Score as a percentage of possible answers to the third question of the sector INF / 0 *“What is a cycle in programming language?”*

**Fig. 10:**
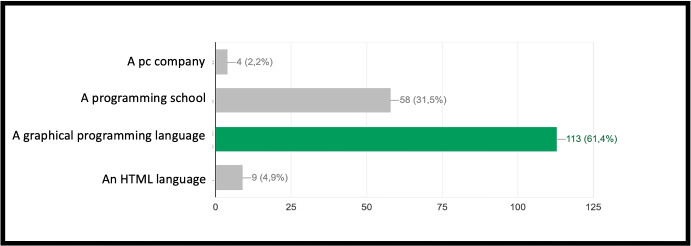
Score as a percentage of possible answers to the 4th of the sector INF/0 *“What is MakeCode?”*

**Fig. 11:**
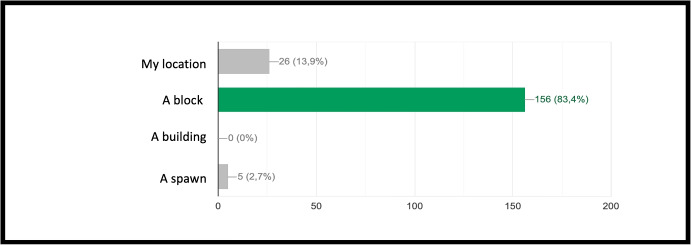
Score as a percentage of possible answers to the first question of the sector GEO / 1 *“What is a point in Minecraft?”*

The first learning area relates to computer knowledge and specific terminology and is identified by the INF / 0 sector code. The first question refers to the use of commands to interface with the game. This evaluates the understanding of the components that make up a computer and therefore the game environment. The second question (which falls within the INF / 0 scope) asks whether it is important, in the context of programming, to assign names to programs inherent to their functionality. The third question relates to the most important concept of programming addressed in the basic course: the syntax of the *cycle*. The first question is investigating the essential understanding of what a cycle is: *“What is a cycle in programming language?”*. There are four options. All of them can cause great confusion in kids who are still new to programming. Learning through gaming, and in this case through Minecraft Education Edition, however, shows that the vast majority of students are able to detect even subtle differences and recognize the most appropriate response. The presence of a game character who positions the blocks one at a time according to the instructions contained in the cycle, allows a solid association between education and its realization, outlining the cause-effect link that is the basis of scientific reasoning. Indeed Fig. 9 shows 95.7% of the students answered correctly and no one answered with the only option which is completely different from the correct answer on a conceptual level. The following question, always linked to the INF field of computer terminology, requires the understanding of what a graphic programming language is: *“What is MakeCode?”.* Figure 10 shows that a very high percentage (61.4 $\%$) of boys answer correctly; however, there is another peak related to the second answer, “A programming school”, which records $31.5\%$. This answer related to the fourth “An HTML language” (percentage $4,9\%$) highlights a reasoning of the students on the composition of the sentence, which leads them to choose, albeit incorrectly, what is closest conceptually to the question posed. Figure 11 instead shows the response percentages assigned to the first question in the geometry sector (GEO / 1). *“What is a point in Minecraft?”*. Geometric reasoning, which has always been in education, requires its concretization through a drawing or the support of objects. The advantage of using software lies in the ability to quickly correct any errors. The advantage of using a game software lies in the possibility of doing it with a playful objective. The very high percentage of positive responses shows how children, through gaming, are able to realize the abstract geometric concept of a point within a defined shape such as the block in Minecraft Education Edition (Figs. 7, 8 and 13).

**Fig. 12:**
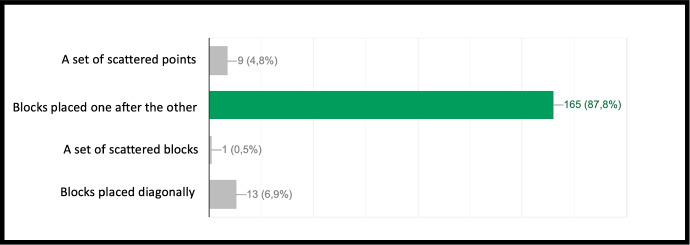
Score as a percentage of possible answers to the first question of the sector GEO/2: *“How is it possible to build a line in Minecraft?*

**Fig. 13:**
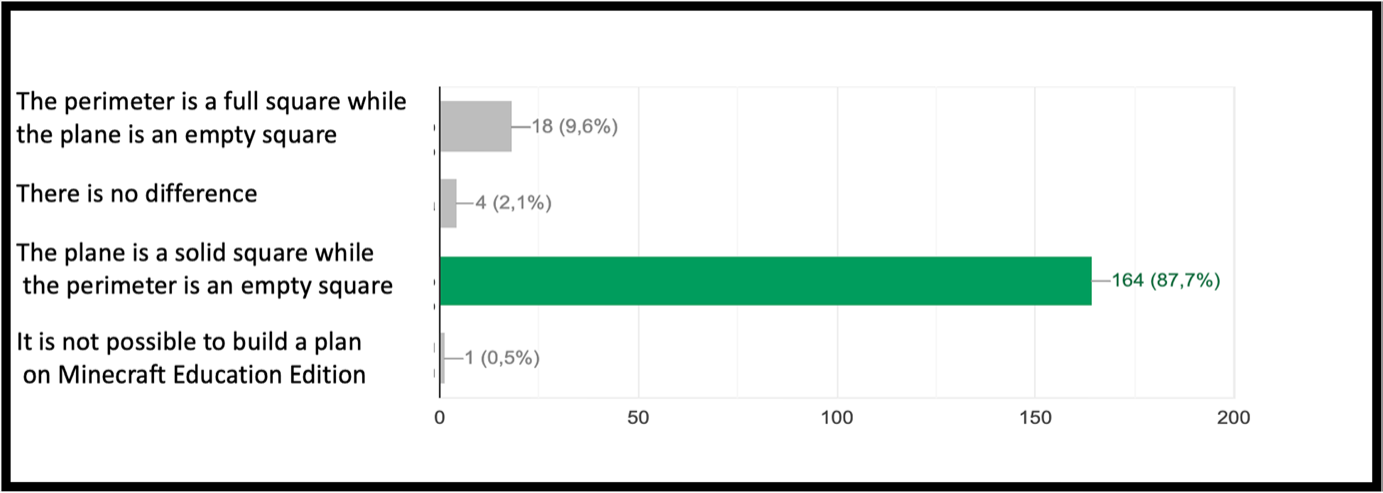
Score as a percentage of possible answers to the first question of the sector GEO/3: *“What is the difference between the plane and the perimeter in Minecraft?”*

**Fig. 14:**
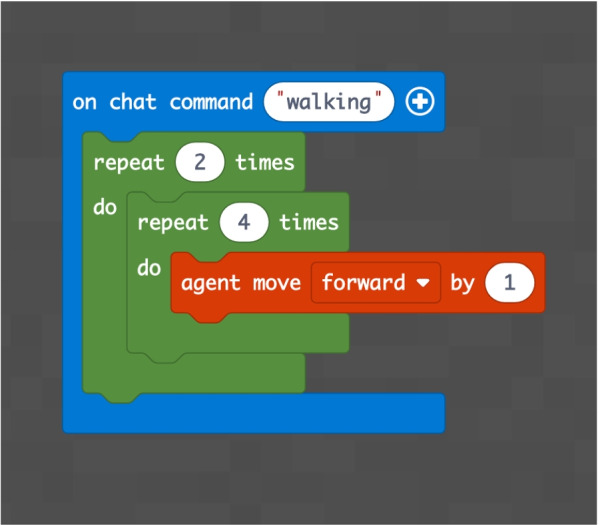
Sector code MAT/1 for the verification of mathematical reasoning skills

**Fig. 15:**
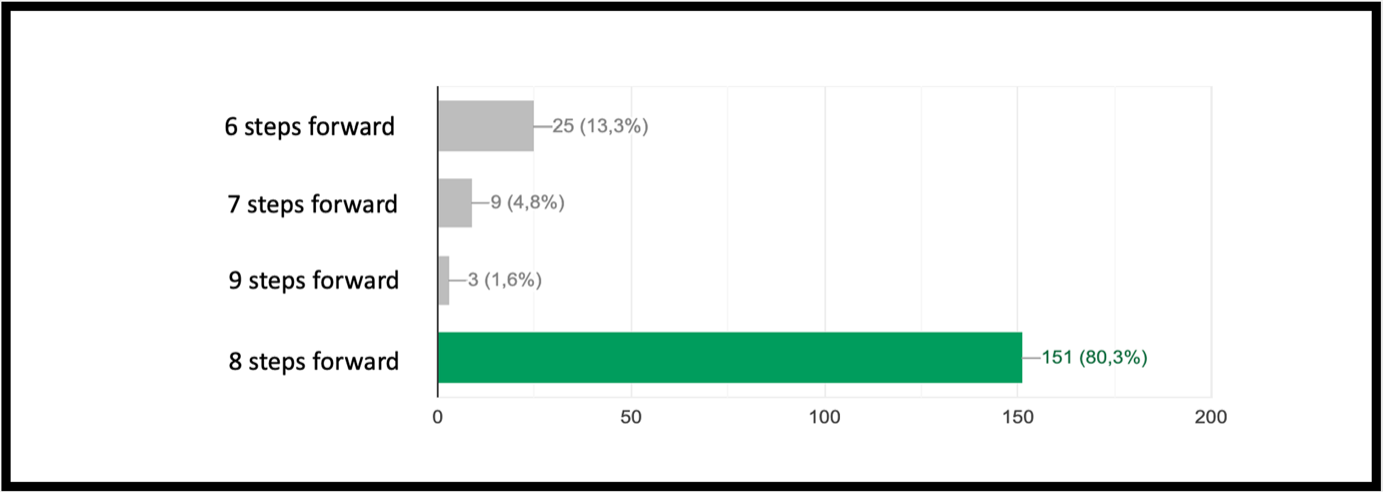
Score as a percentage of possible answers to the question of sector MAT/1: *“How many steps forward does the Agent take?”*

**Fig. 16:**
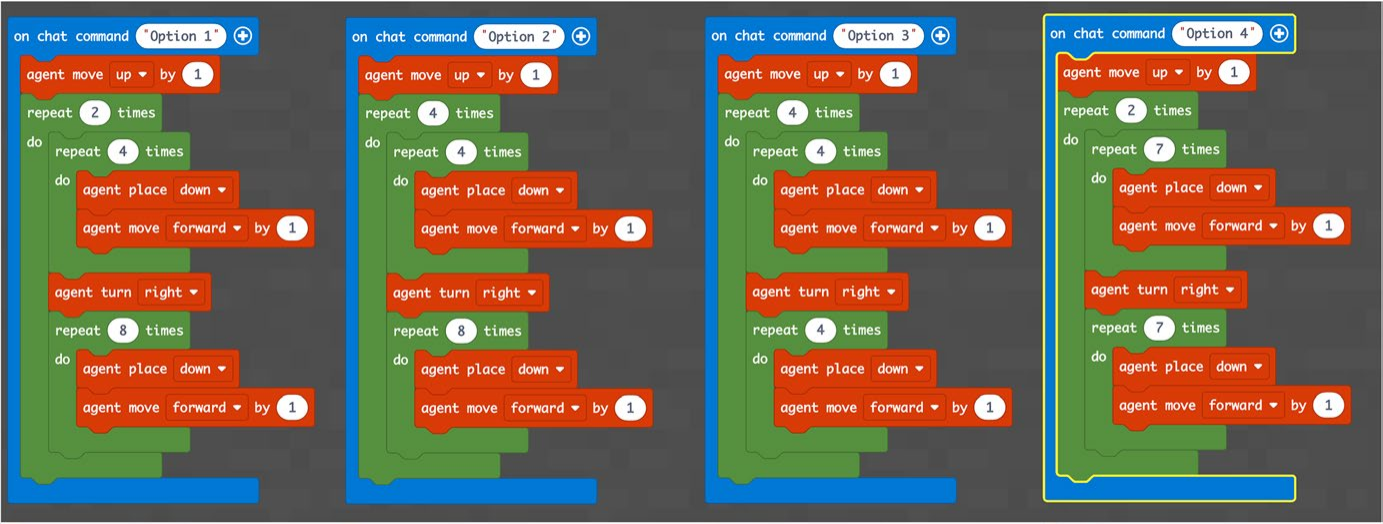
Proposed code for sector INF / 1 related to the question: *“What code builds a rectangle?”*

**Fig. 17:**
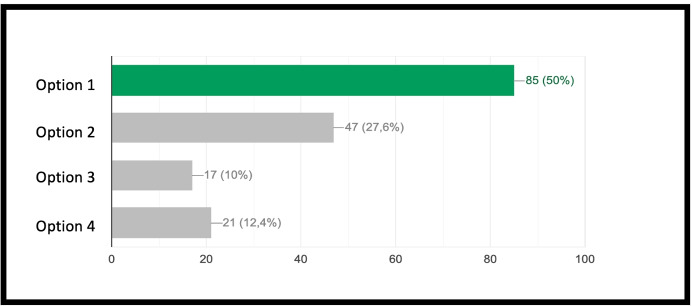
Score as a percentage of possible answers to the question of sector INF/1: *“What code builds a rectangle?”*

**Fig. 18:**
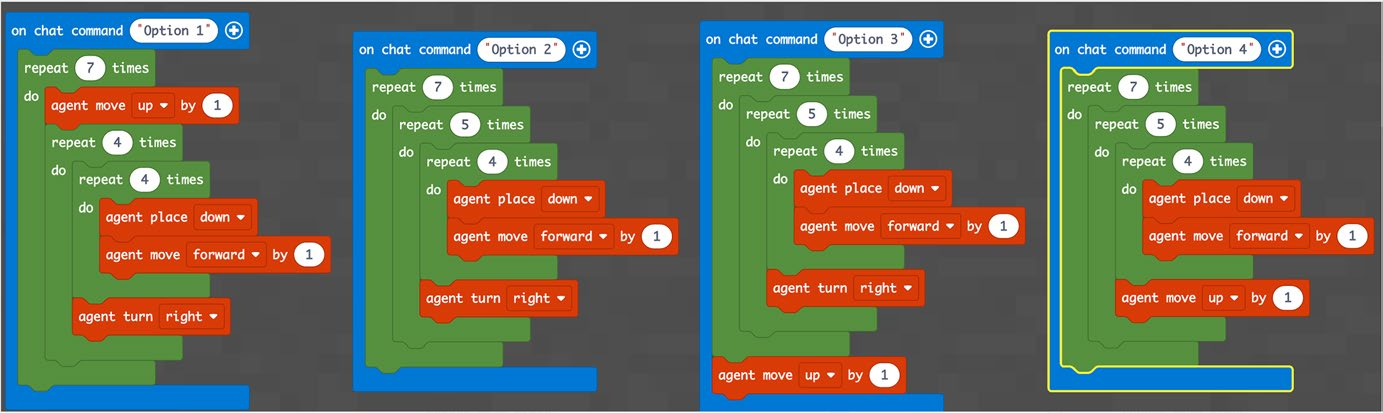
Proposed code for sector INF/2 related to the question: *“What code builds a house?”*

**Fig. 19:**
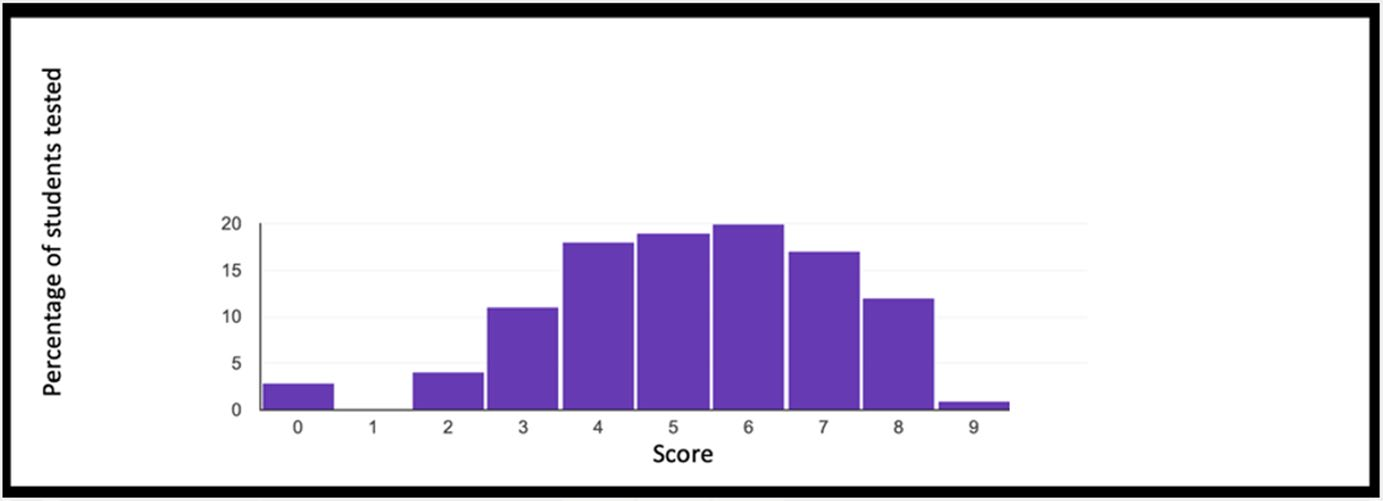
Summary histogram of the scores obtained by students of the basic course in the tests administered

The concept of point (or block in the case of Minecraft) is extremely important because, starting from its definition, it allows to establish all the other properties of the geometry. Similarly, once you understand that a block represents a point in Minecraft geometry, it will be possible to proceed with increasingly complex constructions. The second question of the geometry group (GEO / 2) starts from the previous question to arrive at a more advanced concept: *“How is it possible to build a line in Minecraft?”*. Also in this case the percentage of correct answers is the highest, demonstrating that the visualization of concepts through the game helps the understanding of abstract and complex concepts. The last question (GEO / 3) analyzes a more complex configuration: “What is the difference between the plane and the perimeter in Minecraft?”. The understanding of this question is a demonstration of the ability to know how to divide complex structures into simple parts and to understand that the plan can be seen as a full perimeter. The percentages of the answers given are shown in the Fig. 12. The test also includes questions in the form of proposed MakeCode codes. Figure 12 shows a code that assumes computer knowledge of cycles and mathematical reasoning skills (the question is classified MAT / 1). Most of the guys ($80.3\%$) answer correctly (Fig. 14) by showing that they have fully understood the computer syntactic construction of cycles and nested cycles. Furthermore, they have shown that they have developed the ability to conduct abstract mathematical reasoning. Through the questions of the INF sector, it is possible to investigate the knowledge and reasoning skills of mathematics and geometry through coding as shown in the Figs. 15, 16 and 17 in which the histograms relating to the response percentages for the possible options are shown. Many guys to solve this question ask to implement the codes (symptom of the consciousness of a method of computational investigation). However, the histogram shown in the Fig. 18 shows the answers provided without the aid of any tool. The test ends with a highly complex question that contains knowledge and reasoning of programming, mathematics and geometry. Figure 19 proposes the last question in which 4 different codes are proposed. The student must be able to identify, without being able to execute it, the correct code to build a house, intended as a superimposition of many rectangles. This last question subsumes the previous coding questions within itself. Also in this case a very high percentage of students is able to make the reasoning sufficient to find the correct answer, while the other three options have been chosen without a “preference”

## Discussion

Recent studies show that game-learning is able to rapidly develop numerous cognitive aspects. The work conducted by the group (Zhang et al. 2021) has shown that action games are able to speed up the development of perception and working memory, compared to those which do not use them. Teaching through play and technological tools have shown they know how to emphasize the creative and cognitive abilities of children, especially between the ages of 7 and 12 (Kangas 2010). Furthermore, the playful aspect favors collaboration between students who identify a common learning and investigation objective (Light & Fawns 2003). Digital games are becoming an emerging tool for education, as reported by Tobias et al. (2015), as they can better capture attention and transfer cognitive skills implicitly. Therefore, these can be used for tasks even outside the game. The results collected in this work confirm the previous studies. Children, especially younger (8-11 years), acquire a deep understanding of the knowledge offered through play. The acquired abilities consist also in transforming practical activity lived through the digital experience into a theoretical aspect. Students have shown they are able to conceptualize their experiences, generalize them and reuse personally developed laws depending on the situation. We can therefore speak of learning by transfer: the knowledge acquired through game-learning develops memory and logical abilities that can be decontextualized and reused later. The results are even more positive if we consider that complex topics ranging from programming to mathematics and geometry are dealt with in just 12 hours of lessons. This learning speed is certainly linked to the playful dimension, which favors attention and socialization, but we believe that it is largely due to the possibility of making abstract knowledge concrete, thanks to the possibility of experiencing the situations studied in the digital world. Despite the great results also found through final tests, this study is still limited in comparison with other educational methods that do not involve the use of technology or the playful component. The Digital Education Lab considers their combination as winning in terms of learning, however future quantative studies of comparison with the results achieved through traditional educational methods, will have to be carried out. In this way it will be possible to have wider results, the superposition of which will provide more precise information about the learning parameters of interest such as learning speed, solidity of the information acquired and the ability to decontextualize.

## Conclusions

With the transition to the digital age, education is increasingly making use of new tools for student training. The use of computers helps to visualize abstract concepts, more difficult to assimilate especially for younger students. In particular, game software add playful and social components, favoring an increase in attention and in learning. The game software used was Minecraft Education Edition. The choice was based on two main features of the software, low number of game constraints and the logical mathematical geometric structure of digital reality. This dual articulation favors two aspects of children’s cognitive development. The absence of strong bonds increases the creative spirit while the mathematical-geometric structure at the base of the game favors its logical growth. The results of the tests administered at the end of the course show that a didactic method set up through game-learning is able to favor a solid and rapid learning of abstract concepts which, as is well known, are often more complex if spread with the traditional educational method. Indeed, the method of education reported proposes an experience to understand a concept. While traditional methods generally provide abstract definitions and notions before practical use, education through game-learning with Minecraft Education Edition is based on the experientiality of concepts. Tests show that students are able to go from practical to abstract degree on their own.

The results reported in Section 4 highlight that students of the basic course (mainly aged 8-10 years) are able to solve abstract and complex scientific problems. Figure 19 summarizes the score earned in the final test (considering a point for each correct answer and zero points for each wrong answer), through a histogram. The points range goes from 0 to 9, and is characterized by an average of 6.9 points and a median of 7 points. The results obtained are very positive and show that game learning promotes rapid and strong learning of even complex concepts. Based on this research, we are carrying out new studies on learning through gaming software such as Minecraft Education and learning through music software. Evolution calls digital and education must keep up. The main limitation of this research is the absence of direct (quantitative) comparative analysis with respect to traditional education methods carried out in state schools. This is due to the absence of structured tests within schools that can be used for analyzes of this type. We are currently carrying out collaborations that may lead to obtaining this type of data.
